# Sexual size dimorphism and male reproductive traits vary across populations of a tropical rainforest dung beetle species (*Onthophagus babirussa*)

**DOI:** 10.1002/ece3.9279

**Published:** 2022-09-16

**Authors:** Kai Xin Toh, Sean Yap, Thary Gazi Goh, Nalini Puniamoorthy

**Affiliations:** ^1^ Department of Biological Sciences National University of Singapore Singapore Singapore; ^2^ Institute of Biological Sciences, Science Faculty University of Malaya Kuala Lumpur Malaysia

**Keywords:** dung beetle, reproductive evolution, sexual selection, sexual size dimorphism, Southeast Asia

## Abstract

Sexual size dimorphism (SSD) arises when natural selection and sexual selection act differently on males and females. Male‐biased SSD is rarer in insects and usually indicates strong sexual selection pressure on male body size in a species. Patterns of SSD can also vary between populations of species that are exposed to different environmental conditions, such as differing resource availability and diversity. Here, we investigate intraspecific variation in SSD as well as relative investment in precopulatory (horn length) and postcopulatory traits (sperm length and testes weight) in a tropical rainforest dung beetle *Onthophagus babirussa* across Singapore and Peninsular Malaysia. Overall, three out of four populations displayed significant male‐biased SSD, and SSD was greater in populations with smaller overall body size. Average male body size was similar across all populations while female body size was significantly smaller in Singapore, suggesting that the pronounced SSD may also be due to stronger sexual selection on male body size in Singapore populations. All populations showed significant investment in horns as a weapon likely used in male‐male competition, while postcopulatory traits showed no clear scaling relationship with body size, suggesting a higher priority on precopulatory sexual traits in the mating system of this species.

## Graphical abstract

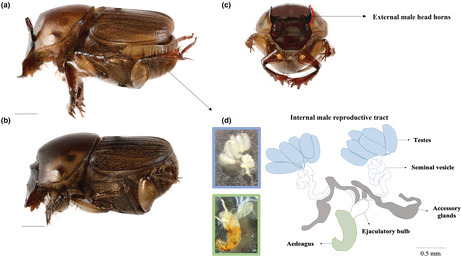

## 1 INTRODUCTION

Sexual selection is defined as selection on heritable traits that vary between individuals within a population that influence reproductive success and fitness (Andersson, 1994). When individuals within a population have differential reproductive success (Panhuis et al., 2001), this can occur prior to copulation (precopulation), when males compete for access to females, leading to evolution of sexual dimorphism in size and secondary sexual traits such as ornaments and weapons (Simmons & García‐González, 2008). Sexual selection can also occur postcopulation, for example, in the form of cryptic female choice, where females can influence the success rate of insemination by males and/or via sperm competition, where sperm from different males compete to fertilize the ova (Birkhead & Pizzari, 2002).

One of the most common traits that is subject to sexual selection is body size. Sexual size dimorphism (SSD) arises when the effects of natural selection and sexual selection act differently on males and females (Blanckenhorn, 2005). In most invertebrates, such as insects, species often display female‐biased SSD, where females are larger due to strong fecundity selection (Blanckenhorn, 2005; Esperk et al., 2007; Rudoy & Ribera, 2017; Stillwell et al., 2010). Larger male body size is usually a derived trait in most insect lineages and an evolutionary reversal of the ancestral state of female‐biased SSD (Blanckenhorn et al., 2004; Blanckenhorn et al., 2007). Most studies on the evolution of male‐biased SSD in insects focus on the effects of intraspecific factors on SSD, such as male–male competition and runaway selection of female‐preferred traits associated with body size (Burkhardt & de la Motte, 1988; Fairbairn & Preziosi, 1994; Pomfret & Knell, 2006; Simmons & Tomkins, 1996; Wilkinson & Reillo, 1994). Fewer studies consider sexual selection in relation to broader ecology, such as external biotic factors. One example would be Beckers et al. (2015) that explored the effect of differential resource competition on divergence in life history traits in separate populations of the dung beetle *Onthophagus taurus*, finding effects of developmental plasticity, parental effects, and genetic background on different traits. In this research, we investigate the differences in SSD and pre‐ and postcopulatory traits in in situ populations of a dung beetle species that differ in resource diversity and availability.

Species belonging to the dung beetle genus, *Onthophagus* Latreille, 1802, (i.e. the most species rich genus in the animal kingdom), have been gaining increased interest as models in evolutionary research. Recent studies show that their morphology and genetic variation can be influenced by sexual selection, parental investment, and environmental variation via a multitude of complex mechanisms (Dury et al., 2020; Hu et al., 2020; Schwab et al., 2019; Snell‐Rood et al., 2016). They are particularly popular in sexual selection research because many species display strong sexual dimorphisms (Parzer & Moczek, 2008). Males often possess horns, a precopulatory sexual trait, on the head and/or thorax, which are used in defending breeding tunnels occupied by females (Garcia‐Gonzalez & Simmons, 2011; Kijimoto et al., 2009; Simmons & García‐González, 2008). Some species exhibit trade‐offs between male horn length and investment in postcopulatory traits such as testes and sperm (Moczek & Nijhout, 2004; Reynolds & Byrne, 2013). Studies in *Onthophagus* have shown alternative mating strategies where smaller males prioritize investing more in testes size and sperm production over horn investment (Simmons & Emlen, 2006; Simmons & García‐González, 2008; Simmons et al., 2007). Sperm length has been shown to be under extreme selection in other insect groups such as in *Drosophila* flies where long sperm are better able to displace sperm from competing males (Lüpold et al., 2016; Snook & Karr, 1998), while shorter sperm has been found to confer higher fertilization success in dung beetles (García‐González & Simmons, 2007). These pre‐ and postcopulatory phenotypes are determined during larval development and affected by the environment and maternal investment such as food provisioning (Emlen, 1994; Emlen, 1997a; Moczek, 1998; Silva et al., 2016). Sexual selection studies of dung beetles often focus on a few model species such as *Onthophagus taurus* (Schreber, 1759), native to the Mediterranean and exotic ranges in Eastern and western North America and Australia, and *O. acuminatus* Harold, 1880, native to Central America (Emlen, 1994; Emlen, 1997a; Moczek, 1998; Silva et al., 2016). More recent studies on *Onthophagus* species from Peninsular Malaysia and Sabah (Goh & Hashim, 2020; Parrett et al., 2019; Parrett et al., 2021; Parrett & Knell, 2018; Pomfret & Knell, 2006) document body size variation but did not report male‐biased SSD among species. Interestingly, surveys conducted in Singapore identified some species where wild‐caught males were consistently larger than females. Of particular interest is the species *Onthophagus babirussa* (Coleoptera: Scarabaeidae; Eschscholtz, 1822), which is widespread across Southeast Asia (SEA; Goh, 2014; Kudavidanage et al., 2012; Priawandiputra et al., 2020; Toh, 2019). Body size of specimens from Peninsular Malaysia appeared similar between the sexes, contrary to specimens collected from Singapore, despite relatively close proximity (~316 km). Intraspecific differences in SSD between separate populations have been observed in other species (Cox & Calsbeek, 2010; Liao et al., 2015; Piross et al., 2019; Rossi & Haga, 2019; Teder & Tammaru, 2005), including a complete reversal of SSD in the dung fly *Sepsis punctum* (Puniamoorthy et al., 2012), and these are usually due to differences in sexual selection pressures acting on each population. Differences in sexual selection pressure can in turn be influenced by external factors such as resource availability (Forsgren et al., 1996; Ghislandi et al., 2018).

In this study, we investigate the variation in SSD and relative investments in pre‐ and postcopulatory traits within and between four separate populations of *Onthophagus babirussa* from Singapore and Peninsular Malaysia (henceforth, SG and MY, respectively). The precopulatory trait examined in this study was male horn length, while testes weight and sperm length were measured as postcopulatory traits. Static allometries were calculated to estimate relative investment in the traits as a function of body size, following standard protocol (Eberhard et al., 2018; Knell, 2009). Resource availability differs between SG and MY since mammal diversity is much higher in the latter. We hypothesize that since dung resources are scarcer and less diverse in SG, competition between males over monopoly of access to dung and females would be higher, and thus male‐biased SSD would be more pronounced in populations from SG than from MY. In line with this, we predict that pre‐copulatory selection acting on SG population is likely stronger than post‐copulatory selection; since male horns are important for male–male combat and mate acquisition (Beckers et al., 2017; Moczek & Emlen, 2000; Simmons & Ridsdill‐Smith, 2011), we hypothesize a greater relative investment in horn length rather than in testes size and/or sperm length.

## 2 MATERIALS AND METHODS

### 2.1 Dung beetle sampling

#### 2.1.1 Study sites

**FIGURE 1:**
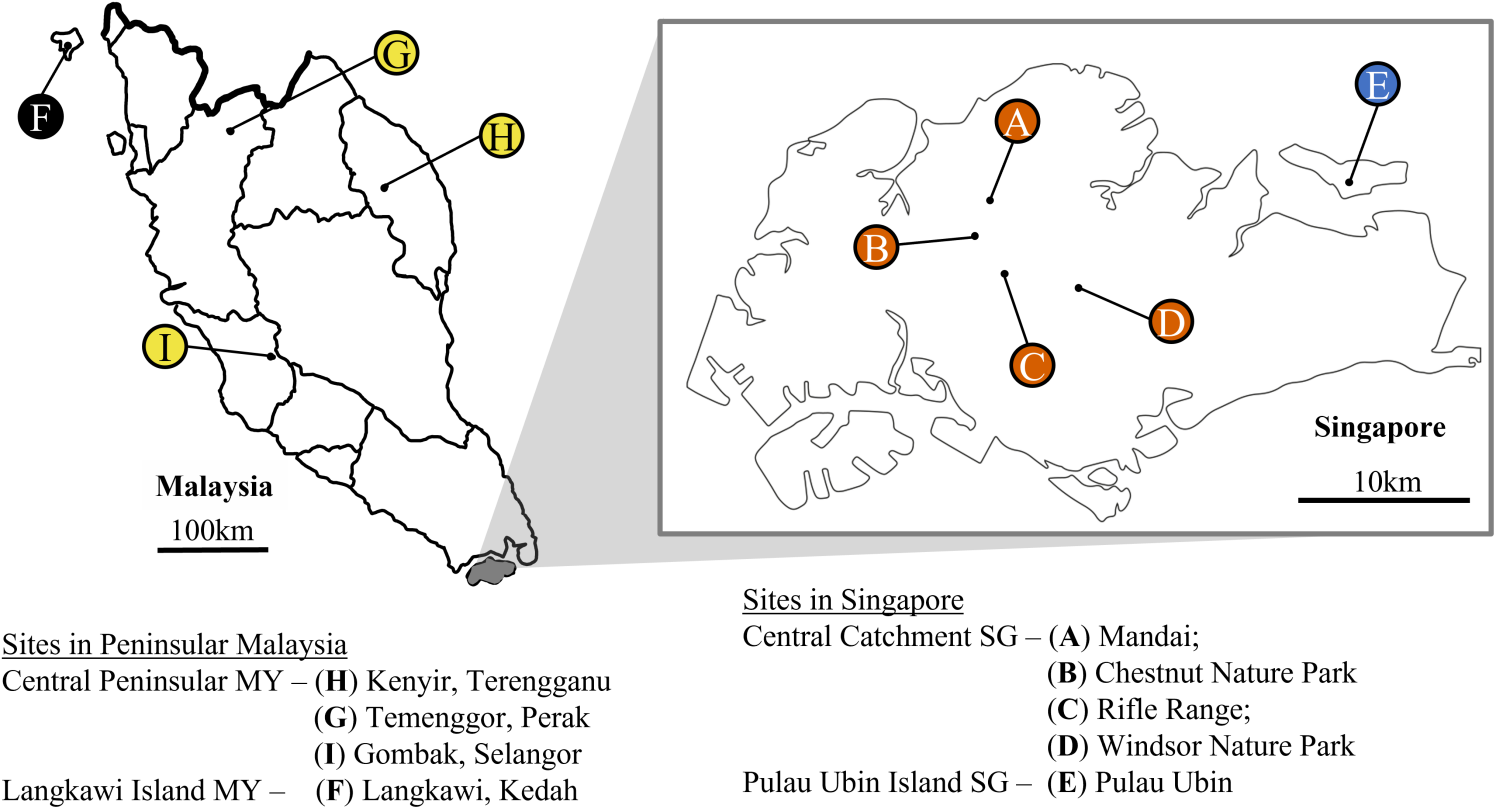
Map of sampling sites located in Singapore and Malaysia. Colours represent the different sites that were treated as separate populations for analyses.

Dung beetles examined in this study were sampled in SG and MY. Figure 1 depicts a map of all sampling sites, and a full list of coordinates is appended in a supplement to the main manuscript (Appendix 1: Table A1). Sampling in SG spanned over 15 months from August 2018 to February 2018 and from May 2019 to December 2019. Specimens from MY were sampled across four sites, Perak, Gombak, Kenyir, and Langkawi, from August to November 2016, as well as in July 2019. Beetles from sites A, B, C, and D were pooled because these sites were part of a continuous stretch of forest in the central region of SG. Pulau Ubin is an island separate from mainland SG and was treated as a population on its own. Sites G, H, and I were pooled as they were all mainland MY sites with connected forests. Langkawi is an island separate from mainland MY and was also treated as its own population. Thus, for all analyses, specimens were separated into four populations—Central Catchment Nature Reserve on mainland SG (Central Catchment SG), Pulau Ubin (Pulau Ubin Island SG), central MY (Central Peninsular MY), and Langkawi (Langkawi Island MY). Literature search was conducted to compile a checklist of non‐volant mammal species present in each of the four study sites with species body size and consumer type, and these are presented and summarized in the appendix (Appendix 1: Tables A2 and A3).

#### 2.1.2 Sampling and sorting protocol

**FIGURE A1:**
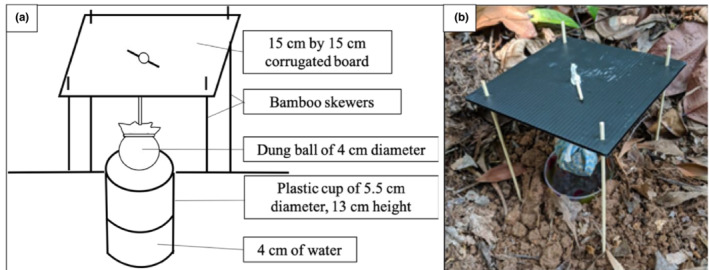
(a) Diagram and (b) photograph showing structure and components of dung‐baited pitfall traps. Human dung was wrapped in cloth to form a “dung ball” of 4 cm in diameter and suspended approximately 4 cm above a buried plastic cup using cotton twine and a shelter made of 15 cm by 15 cm corrugated board. Buried plastic cups were filled with 4 cm of water to wet the wings of dung beetles that have fallen in to prevent the beetles from escaping.

Dung beetle sampling was conducted using baited pitfall traps and baited funnel pitfall traps with human dung as the bait because it is widely accepted to be the best bait to attract a wide variety dung beetles (Howden & Nealis, 1975; Kudavidanage et al., 2012; Larsen & Forsyth, 2005). Exact details of trap materials and construction are appended (Appendix 1: Figure A1). Traps were retrieved after 24–48 h, and captured beetles were brought back to the laboratory for morphological identification and sorting using an Olympus SZX10 microscope.

**FIGURE A2:**
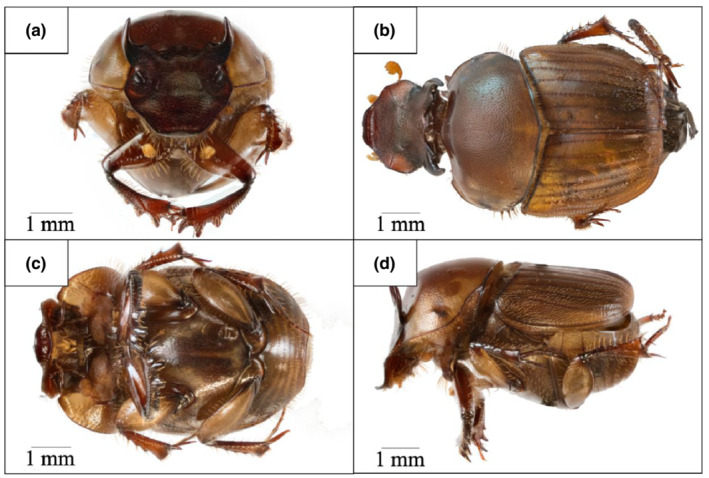
Images of male *O. babirussa* in four angles (a) Anterior (b) Dorsal (c) Ventral (d) Lateral.

**FIGURE A3:**
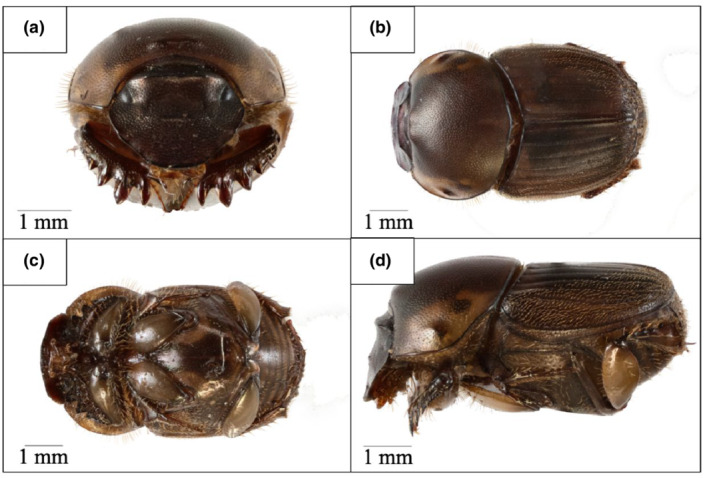
Images of female *O. babirussa* in four angles (a) Anterior (b) Dorsal (c) Ventral (d) Lateral.

**FIGURE A4:**
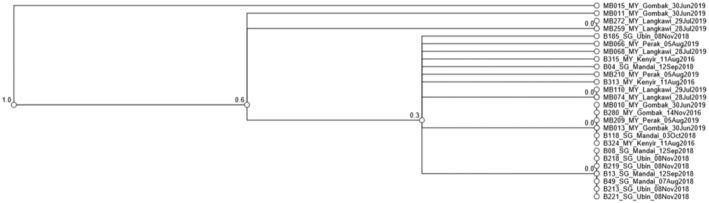
Cluster fusion diagram constructed based on uncorrected pairwise distances between COI barcode sequences from 26 representative specimens from across the main sampling sites.

*Onthophagus babirussa* were separated from other species via sorting by morphological characters (see Appendix 2: Figures A2 and A3) and DNA barcoding. Specimens used for DNA barcoding were killed and preserved in 70% molecular grade ethanol. DNA was extracted from 739 specimens from Singapore populations (CCNR = 129 and Pulau Ubin = 167) and Malaysian populations (Central Peninsular MY = 109 and Langkawi = 334). For these specimens, the right mid femur was dissected into 7 μl of QuickExtract solution, and the DNA was extracted by following the manufacturer's protocol (Lucigen, 2018). Then, 313 bp fragments of the COI gene were amplified via PCR (see Appendix 2 for detailed protocol), sent for next‐generation sequencing (NGS) and used for DNA barcoding. Sequence analysis was then conducted with reference to the analysis pipeline detailed by (Meier et al., 2016), and a well‐established 3% threshold for uncorrected pairwise distances was used to delimit different species (Hebert et al., 2003; Meiklejohn et al., 2011; Srivathsan & Meier, 2012). All specimens examined in this study fell within the same molecular cluster under this 3% threshold, and a cluster fusion diagram with representatives from each population is appended in Appendix 2 (Figure A4), along with the full protocol for morphological and molecular sorting. The molecular barcodes were congruent with our morphological sorting and general consensus with the geographical sampling.

### 2.2 Documenting reproductive trait variation

#### 2.2.1 Precopulatory trait measurements

**FIGURE 2:**
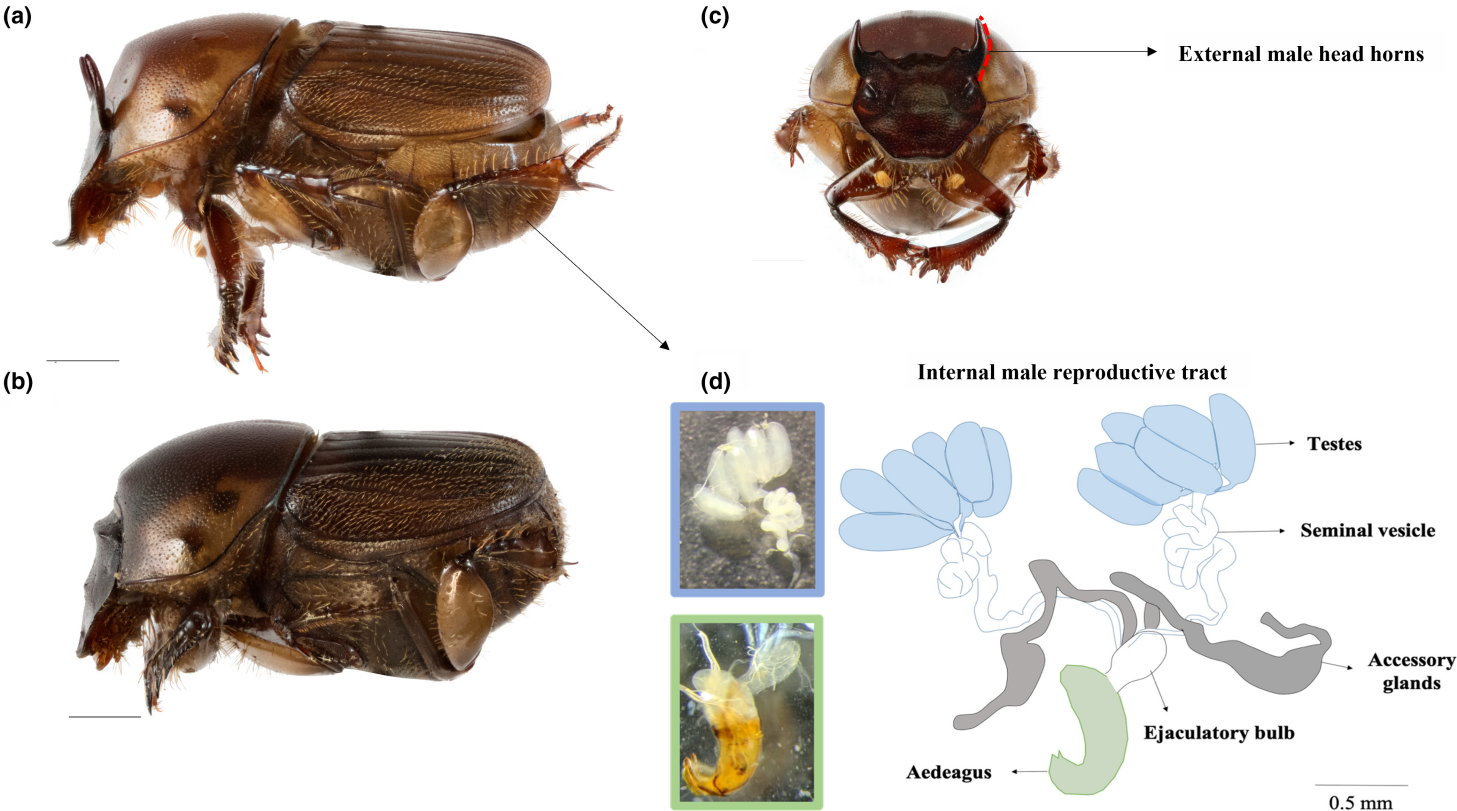
Precopulatory (horn length and maximum pronotum width) and postcopulatory traits (sperm length and testes weight) were measured in male *O. babirussa;* (a) lateral view of adult male; (b) laternal view of adult female; (c) front view of male with red trace on head horn; (d) drawing of male reproductive tract with the pictures of the testes and seminal vesicles (blue insert) and aedaegus (green insert).

To investigate the sexual size dimorphism in the four populations of *O. babirussa*, maximum pronotum width (Figure 2) of males and females was measured as a proxy for body size with the eyepiece reticle on the Olympus SZX10 microscope. This is widely used as a proxy for body size because the pronotum width does not change in adulthood and has been found to be the most appropriate measure for body size in dung beetles (Emlen, 1997a, 1997b; Knapp & Knappová, 2013).

Horn lengths of male *O. babirussa* (Figure 2) were measured to document variation in this precopulatory trait. Images were taken of the anterior habitus. Heads of the beetles were separated and suspended with Durex KY Jelly, with horns aligned parallel to the lens of the camera. Images were captured using the EOS 800D and 6D camera body with the Canon MP‐E 65 mm f/2.8 1‐5× lens at 5× optical zoom. The camera was suspended on the Dun, Inc. P‐51, and the Camlift controller V2.9.3.0 software was used to take multiple images at different heights for focus stacking. EOS Utility Launcher software was used to access the images and stack them using the Zerene Stacker V. 1.04. software. Stacked images were imported to Adobe Photoshop CS5 V. 12.0 ×64, and a 1 mm scale bar was added to each image. Next, processed images were imported to ImageJ V. 1.51, and the horns were measured from the tip to the bottom of the outer edge of each horn, following previous studies (Moczek & Emlen, 1999).

#### 2.2.2 Postcopulatory trait measurements of male specimens

Abdomens of male *O. babirussa* specimens were dissected into 1× phosphate‐buffered solution (PBS) to measure the following postcopulatory traits: testes weight and sperm length (Figure 2). Testes were isolated and transferred onto pre‐weighed aluminium sheets and dried in a Memmert Gravity Basic Digital Oven D overnight. Then, total weight was measured on the Mettler Toledo ML104 Newclassic ml Analytical Balance. Weight of the testes was calculated by subtracting the weight of the aluminium sheet from the total weight.

To measure the sperm length, seminal vesicles containing the mature sperm were first isolated and transferred onto a drop of PBS on a frosted slide. Then, sperms were teased out from the vesicles using an insect pin. Slides were dried in the oven, and sperms were fixed onto the slides with a solution of three parts methanol and one part acetic acid for 2 min. Next, the slides were washed in 1× PBS for 1 min, and the sperms were stained for 5 min in the dark with 4′,6‐diamidino‐2‐phenylindolev (DAPI), which binds to DNA to form a fluorescent complex to allow for visualization of sperm heads under a fluorescent microscope. Following that, the slides were washed in 1× PBS and placed in the dark to dry. When the slides were dried completely, one to two drops of glycerol were added on the stained regions, coverslips were placed, and the edges were sealed with clear nail polish and left to dry in the dark. The sperms were visualized using an Olympus BX50 fluorescence microscope and measured using μManager and ImageJ V. 1.51 software. Based on previous studies, 5–10 sperms were measured per specimen (García‐González & Simmons, 2007; Simmons & Kotiaho, 2002; Werner & Simmons, 2011).

### 2.3 Statistical analyses

Box plots of average pronotum width were constructed with confidence intervals using the R packages *ggplot2* (Wickham, 2016), *dplyr* (Wickham et al., 2020), and *plotrix* (Lemon, 2006) and tested for significance in body size difference between the sexes within each population using ANOVA, checking the residuals for normality after. To test if SSD varied between populations, we ran linear models testing for significant sex by location interaction. Post‐hoc analyses using Dunn test were also conducted to determine which populations differ from the other for male and female body size. In addition, the sexual dimorphism index (SDI) was calculated for each population following the formulation by Lovich and Gibbons (1990), where the mean size of the larger sex is divided by the mean size of the smaller sex. A negative sign is arbitrarily added to the SDI as the males are larger (Lovich & Gibbons, 1990).

**FIGURE 3:**
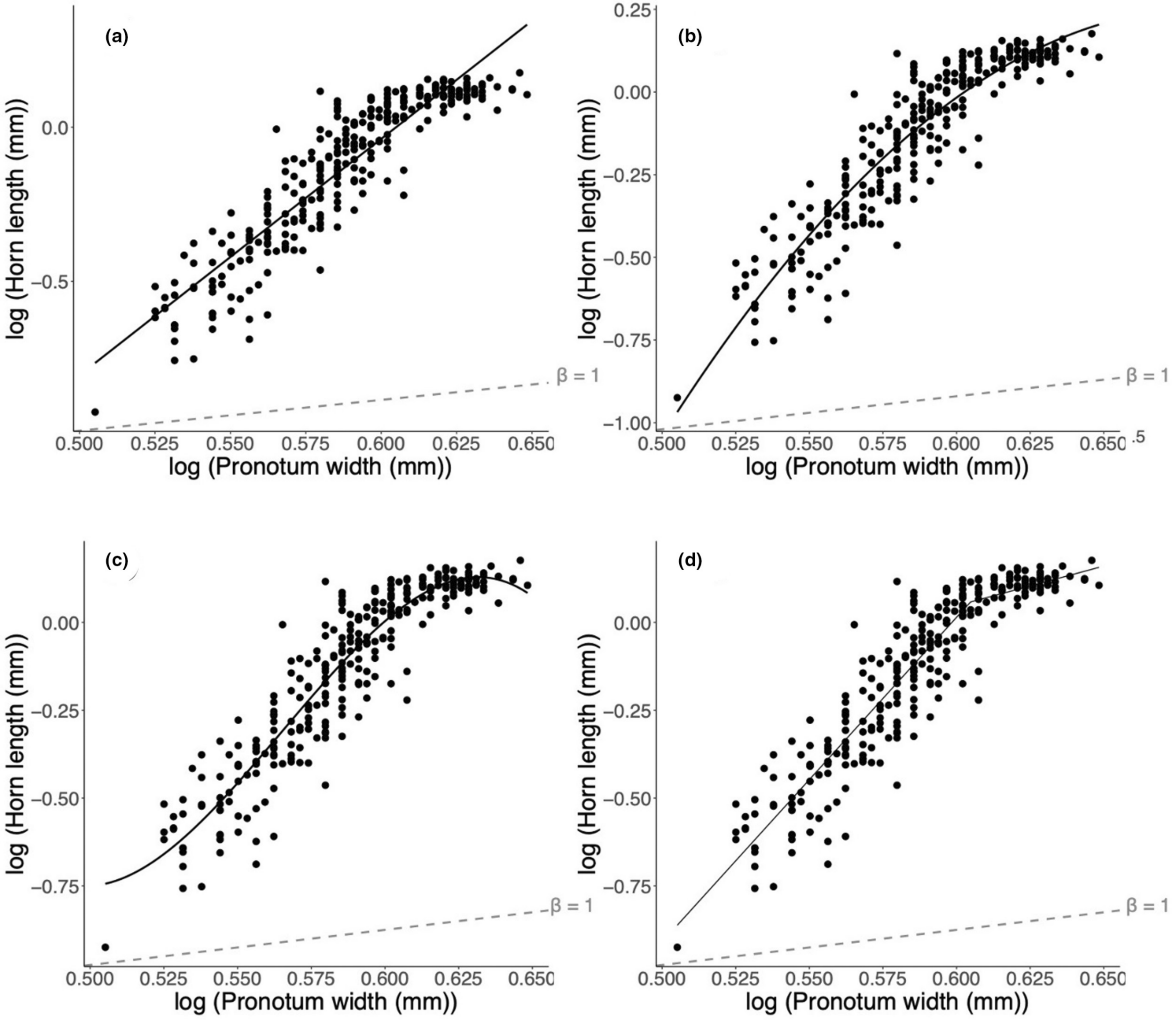
Log–log scatterplot to determine the allometric relationship between horn length and body size (pronotum width) in male *O. babirussa* from Singapore. Following recommendations by Knell (2009), we fitted (a) linear model, (b) quadratic model, (c) cubic model, and (d) breakpoint model using the R package segmented (Muggeo, 2008) to the pooled data with all four populations to characterize the horn length‐body size (*n* = 292).

**FIGURE A5:**
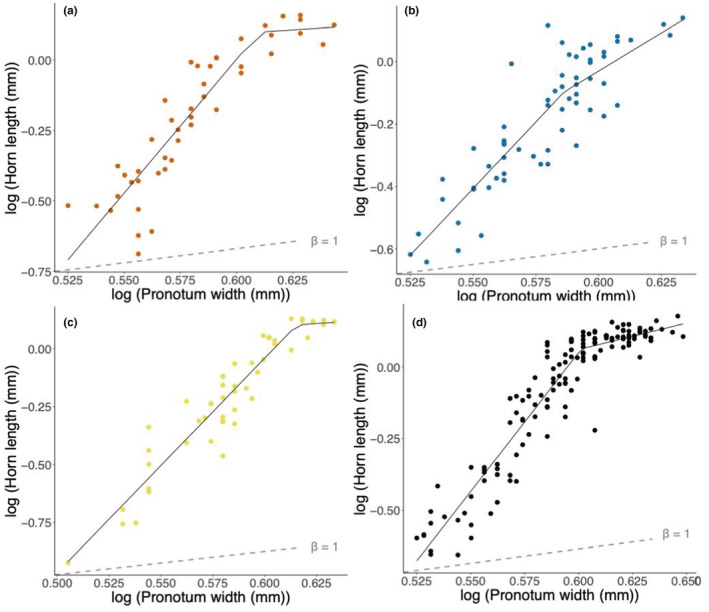
log–log scatterplot to determine the allometric relationship between horn length and body size (pronotum width) in male *O. babirussa* from (a) Central Catchment SG (*n* = 45), (b) Pulau Ubin Island SG (*n* = 61), (c) Central Peninsular MY (*n* = 46) and (d) Langkawi Island MY (*n* = 139), using the breakpoint model.

To determine whether populations differed with respect to relative investments in precopulatory and postcopulatory traits, the static allometries were calculated by first constructing log–log scatterplots of trait size against pronotum width. As the log–log scatter plot of horn length against pronotum width displayed a clear nonlinear relationship, we followed the recommendations by Knell (2009) and Parrett et al. (2021) and fitted (1) linear model, (2) quadratic model, (3) cubic model, and (4) breakpoint model using the R package *segmented* (Muggeo, 2008) to the pooled data with all four populations to characterize the trait size–body size relationship Figure 3. Model selection was then conducted with the Akaike information criterion (AIC). The breakpoint model had the lowest AIC score for horn length (Table 1), indicating that this model is the best model for explaining the relationship between the variables (Knell, 2009). Following this, allometries were also calculated for the overall data separated by (1) population and (2) minor or major morphs as determined by the breakpoint models applied to each population (see Appendix 3: Figure A5).

**TABLE 1:** Akaike information criterion (AIC) to compare the four models fitted for horn allometry

Type of model	*df*	AIC	ΔAIC
Linear model	3	−507.8343	61.9236
Quadratic model	4	−548.6784	21.0795
Cubic model	5	−569.5630	0.1949
Breakpoint model	5	−569.7579	0

## 3 RESULTS

### 3.1 Variation in sexual size dimorphism (SSD)

**FIGURE 4:**
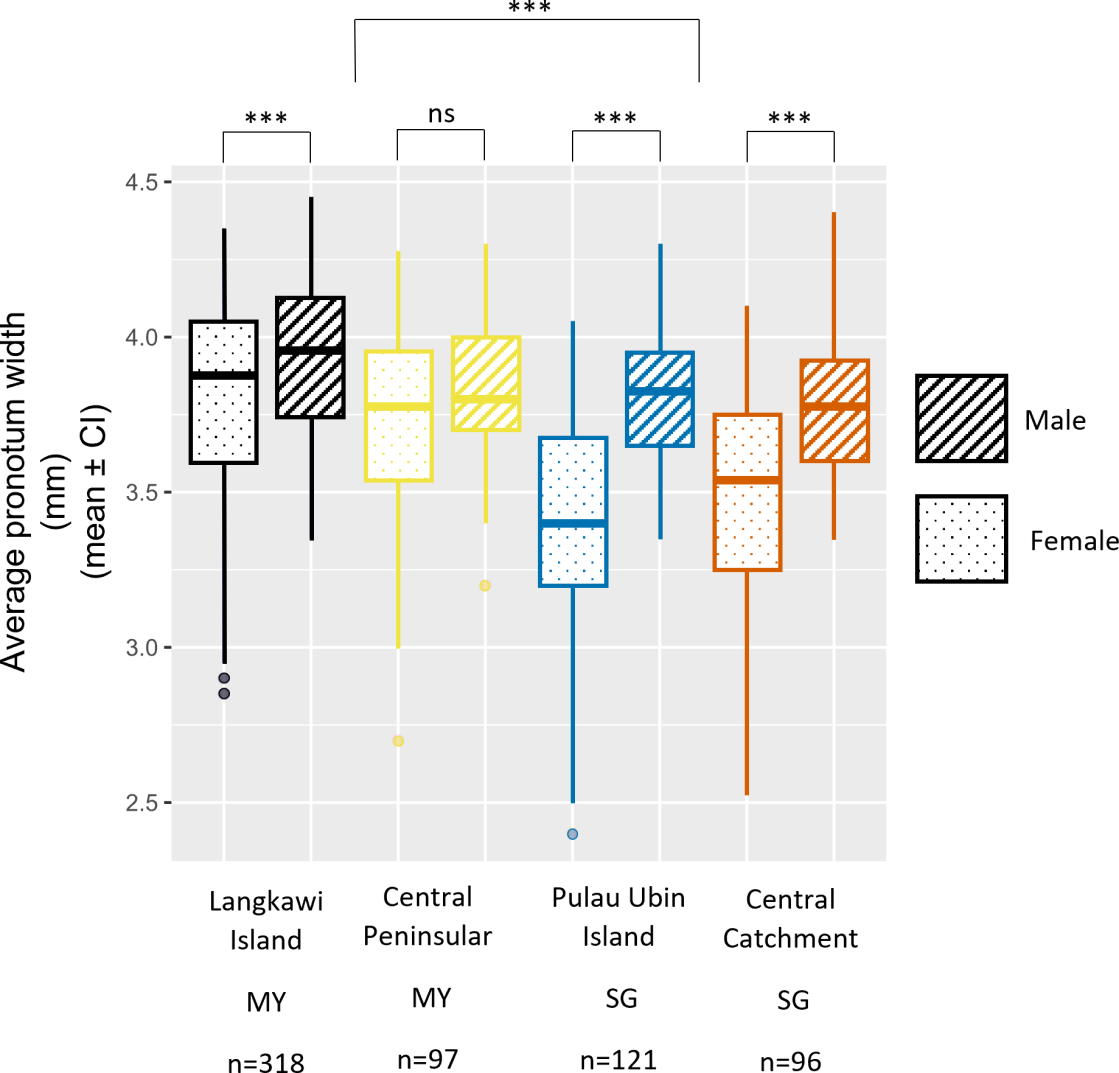
Comparison of the average body size (pronotum width) of female and male *O. babirussa* across populations from Malaysia and Singapore to determine the presence of sexual size dimorphism (SSD). SSD varied across populations (***p < .001, ns = not significant).

To test if SSD varied between populations, we ran linear models and found that the best fitted model with normally distributed errors included significant sex by location interaction, showing that SSD differed between populations (Table 2). Males were significantly larger than females in both SG populations (ANOVA: Central Catchment SG: *p* < .0001, Pulau Ubin Island SG: *p* < .0001) and Langkawi Island MY (ANOVA: *p* < .0001), indicating a clear male‐biased SSD (Figure 4). Although males in Central Peninsular MY were also larger than females, this difference was not statistically significant (ANOVA: *p* > .05). In addition, the SDI was more pronounced in SG populations (Central Catchment SG = −1.09, Pulau Ubin Island SG = −1.12; Central Peninsular MY = −1.03 and Langkawi Island MY = −1.03), even though the average body size of males in MY populations is bigger than that of Singapore population.

**TABLE 2:** Akaike information criterion (AIC) to compare the linear models (lm) testing the effects of sex and locality on body size

Model	*df*	AIC
Body Size ~ 1	2	440.0202
Body Size ~ Sex	3	391.7405
Body Size ~ Locality	5	375.2898
Body Size ~ Sex + Locality	6	313.2744
Body Size ~ Sex * Locality	9	292.3150

Females from MY populations were significantly larger than females from SG populations while males from Langkawi Island MY were significantly larger than males from SG populations but did not differ significantly with Central Peninsular MY (Table 3). Body size also did not differ significantly between the SG populations and Central Peninsular MY (Table 3).

**TABLE 3:** Summary of body size differences between populations following post‐hoc Dunn's test (***p < .0001, **p < .01, ns = not significant [*p* > .5]), divided by sex where blue cells refer to males and red cells refer to females

	Central catchment SG	Pulau Ubin Island SG	Central peninsular MY	Langkawi Island MY	
Central Catchment SG		ns	ns	MLKMY > CCSG**	
Pulau Ubin Island SG	ns		ns	LKMY > PUSG**	Male
Central Peninsular MY	CPMY > CCSG***	CPMY > PUSG ***		ns	
Langkawi Island MY	LKMY > CCSG***	LKMY > PUSG***	ns		Female

### 3.2 Variation in male reproductive traits as a function of body size

**FIGURE 5:**
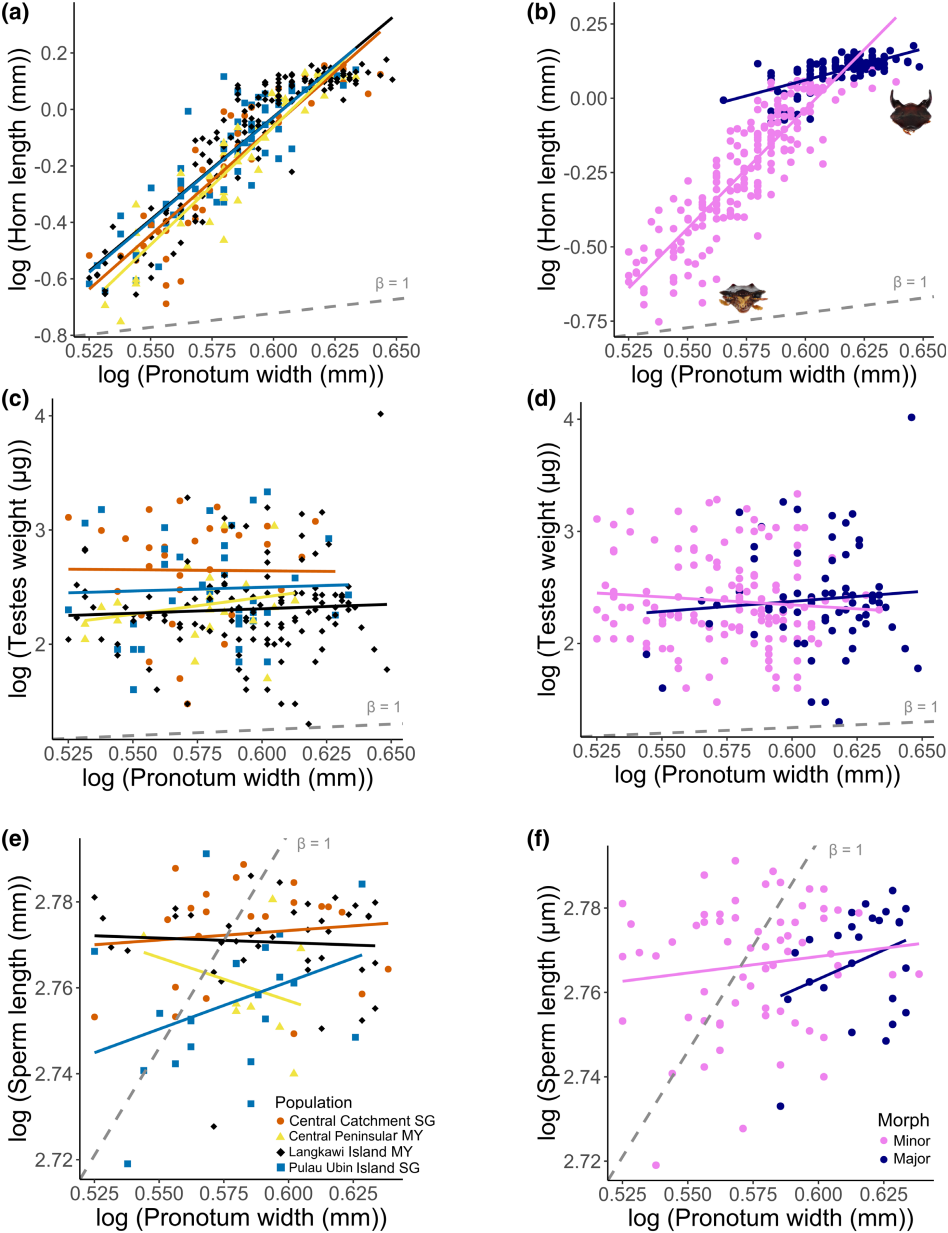
Log–log scatterplots to determine allometric relationship between body size and horn length (overall *β* = 7.5) by (a) population (central catchment SG: *n* = 45, *β* = 7.7; central peninsular MY: *n* = 45, *β* = 8.3; Langkawi Island MY: *n* = 138, *β* = 7.3; Pulau Ubin Island SG: *n* = 61, *β* = 7.3), and (b), minor (*n* = 196, *β* = 8) and major (*n* = 93, *β* = 2.1) morphs; body size and testes weight (overall *β* = −0.3) by (c) population(central catchment SG: *n* = 31, *β* = −0.2; central peninsular MY: *n* = 21, *β* = 3; Langkawi Island MY: *n* = 122, *β* = 0.8; Pulau Ubin Island SG: *n* = 37, *β* = 0.6), and (d) minor (*n* = 136, *β* = −1.4) and major (*n* = 70; *β* = 1.8) morphs; body size and sperm length (overall *β* = 0.1) by (e) population (central catchment SG: *n* = 22, *β* = 0.04; central peninsular MY: *n* = 8, *β* = −0.2; Langkawi Island MY: *n* = 39, *β* = −0.02; Pulau Ubin Island SG: *n* = 21, *β* = 0.22), and (f) minor (*n* = 65; *β* = 0.1) and major (*n* = 25; *β* = 0.3). Dashed gray lines show the isometric line (*β* = 1).

Using a log‐transformed data and the breakpoint model, a hyperallometric relationship (allometric coefficient, *β* > 1, Figure 5a, Table 4) was found between horn length and body size (pronotum width) for all four populations of *O*. *babirussa*. The adjusted *R* ^2^ values for equation one of the breakpoint models were high for all four populations, signaling a strong positive correlation. In addition, 95% confidence intervals (CIs) for equation 1 of all populations excluded zero, ruling out the likelihood of a zero slope, indicating a significant relationship between horn length and body size. These results suggest that body size is a significant factor in explaining horn length variation, where larger males have disproportionately longer horns. Interestingly, there is an overlap in CI values for all populations, which suggests that there were no significant population‐level differences in allometric relationships (Figure 5a, Table 4). Overall analysis of horn length allometry separated by morphs found that both morphs showed hyperallometry, but minor morphs showed greater investment (*β* = 8) than major morphs (*β* = 2.1; Figure 5b).

**TABLE 4:** Summary of allometric coefficients and model outputs for horn length, testes weight, and sperm length allometry

Population	Statistical model	Allometric coefficient	Adjusted *R* ^2^	95% confidence interval	*p*‐Value
Horn length allometry					
CCNR	Breakpoint	9.500	0.824	[7.628, 11.372]	NA
Pulau Ubin	Breakpoint	8.552	0.766	[6.633, 10.471]	NA
Central Peninsular MY	Breakpoint	9.265	0.889	[8.132, 10.398]	NA
Langkawi	Breakpoint	9.685	0.899	[8.895, 10.476]	NA
Testes weight allometry					
CCNR	Linear	−0.189	−0.034	[−7.360, 6.983]	0. 957
Pulau Ubin	Linear	0.647	−0.027	[−5.187, 6.481]	0.823
Central Peninsular MY	Linear	2.977	0.001	[−3.203, 9.156]	0.326
Langkawi	Linear	0.786	−0.005	[−1.633, 3.204]	0.521
Sperm length allometry					
CCNR	Linear	0.044	−0.039	[−0.151, 0.239]	0.643
Pulau Ubin	Linear	0.220	0.078	[−0.060, 0.501]	0.117
Central Peninsular MY	Linear	−0.201	−0.063	[−0.845, 0.443]	0.474
Langkawi	Linear	−0.022	−0.023	[−0.136, 0.093]	0.705

On the contrary for postcopulatory traits, using log‐transformed data, investments in both testes weight and sperm length increase somewhat, but the 95% confidence intervals overlap with both zero and unity (Figure 5c,e, Table 4). Thus, this increase in investment is not significant, and neither trait significantly deviates from isometry either, showing no clear relationship between body size and the measured postcopulatory traits. Splitting the data for both postcopulatory traits by minor and major morphs showed higher allometric values for major morphs, which does not suggest a trade‐off between body size and investment in postcopulatory traits as seen in some other dung beetle species (Figure 5d,f). Testes weight showed negative allometry in minor morphs (*β* = −1.4) and slight hyperallometry for major morphs (*β* = 1.8), while sperm length showed hypoallometry for both minor (*β* = 0.1) and major (*β* = 0.3) morphs.

## 4 DISCUSSION

### 4.1 Sexual size dimorphism (SSD) varied among populations

Overall, our results showed that there is significant male‐biased SSD in all populations except Central Peninsular MY, and that there is significant investment in precopulatory weapons, but no clear trend observed regarding investment in postcopulatory sexual traits.

Since mammals are of lower abundance and diversity in Singapore's forests, dung resources in Singapore are scarcer and less diverse, possibly leading to greater competition between males and higher sexual selection pressure. We thus hypothesized that male‐biased SSD would be greater in Singapore than Peninsular Malaysia, and our results mostly agree with the hypothesis. Before discussing SSD, however, we first must address the finding that average body size and specifically female body size were found to be much smaller in both SG populations as compared with both populations from MY. This disparity could be due to weaker fecundity selection on females in SG. We had planned to study this by examining female fecundity via measuring the spermathecae or rearing females and measuring clutch sizes, but we were unable to obtain enough data for either. Future common garden experiments with lines from wild‐caught females from the four populations should be carried out to determine if populations differ in fecundity between SG and MY. If female beetles from SG are found to produce smaller clutch sizes, this could provide evidence for lower fecundity in SG populations that could explain the smaller female body size. Another possible reason that could lead to smaller body size is viability cost. Larger body sizes require greater resource consumption during development, longer development times, and higher energy requirements during both development and adulthood, all of which could be especially detrimental in an environment where resources are scarce. In resource‐scarce SG, females may have to prioritize mating and offspring production opportunity over clutch size. Females would thus benefit from faster development time that usually results in smaller body sizes, as early maturation affords more mating opportunities. Smaller body sizes also reduce energy requirements, possibly allowing females to afford more time to mating rather than foraging.

Even though females from SG were much smaller, males were about the same size as their MY counterparts, emphasizing the strong male‐biased SSD in SG. Our documentation of significant male‐biased SSD in SG and Langkawi Island is interesting since it is a rare trait in this taxon. In beetles, only 9% of the reported species exhibit male‐biased SSD while 72% exhibit female‐biased SSD (Stillwell et al., 2010). More specifically, most *Onthophagus* species do not display sexual size dimorphism (Pomfret & Knell, 2006). In fact, a study of six Southeast Asian *Onthophagus* species, including *O. babirussa* (from MY), reported no SSD (Goh & Hashim, 2020). Our results were concordant with this study, showing that there was indeed no significant SSD among specimens from MY. However, populations of the same species from Central Catchment SG, Pulau Ubin Island SG, and Langkawi Island MY all showed significant male‐biased SSD (Figure 4).

Theory suggests that strong precopulatory sexual selection drives male‐biased SSD in insects as larger body size in males has been widely documented to increase mating success due to female choice or male–male competition (Blanckenhorn, 2005; Puniamoorthy et al., 2012; Stillwell et al., 2010). In many *Onthophagus* dung beetles and related taxa, males compete to gain access to females and body size is a predominant factor in determining fighting success (Emlen, 1997a, 1997b; Moczek & Emlen, 1999). However, the intensity of sexual selection acting on male body size is not necessarily stronger than the fecundity selection acting on female body size. In beetles, larger females are generally able to produce larger and more offspring, thus accounting for the female‐biased SSD observed in most species (Stillwell et al., 2010). As such, the male‐biased SSD in *O. babirussa* is likely a derived trait that can be due to a relative increase in the intensity of sexual selection on male body size in this species. Our results also showed a strong investment in horns, a precopulatory weapon, further supporting that strong sexual selection is acting on males in this species via male–male competition.

One possible factor that could contribute to both lower female fecundity and stronger sexual selection on males in SG is resource availability, specifically dung resource. In SG, approximately 95% of forests were cleared over the last 200 years due to urbanization, causing high local extinctions of fauna such as birds and mammals in forest habitats (Bickford et al., 2010; Brook et al., 2003). Singapore's remaining forests are mostly degraded, highly fragmented, and often subjected to high levels of disturbances, leading to a decrease in the general abundance of mammals (Bickford et al., 2010; Lee et al., 2009). Thus, there are fewer food and brood resources, leading to fewer opportunities for oviposition in female dung beetles in SG where the main sources of dung are likely from long‐tailed macaques (*Macaca fascicularis*) and wild boars (*Sus scrofa*; Culot et al., 2013). It is possible that domestic animals such as feral dogs and cats could also contribute dung resources in Singapore's urban context, but the numbers of these non‐native domestics have been greatly reduced due to government efforts, and surveys of SG's urban areas only found a few resilient species of dung beetles that did not include *O. babirussa*. Singapore's domestic mammal farming industry is also nearly nonexistent, with only a handful of remaining farms located in the northwest. The closest other source of abundant and diverse mammal dang would be in the Mandai area in the proximity of the Central Catchment area in which we surveyed, where the Singapore Zoo and other Mandai wildlife attractions are located, but even then mammal abundances are not high. In contrast, the sites surveyed in MY are located within larger stretches of forests that serve as a refuge for larger mammals not found in SG. Comparing mammal diversity, mainland peninsular Malaysia has more than three times the number of non‐volant mammal species than mainland Singapore (Appendix 1: Tables A2 and A3). Many taxa that contribute large diversity and volumes of dung resource that are present in MY are absent from SG, including most large herbivores such as the Asian Elephant (*Elephas maximus*), Malayan Tapir (*Tapirus indicus*), and the Bovidae family, as well as large carnivores such as the Tiger (*Panthera tigris*) and Clouded Leopard (*Neofelis nebulosa*). The presence of more and larger species provides female dung beetles more food and brood resources for oviposition opportunities (Qie et al., 2011; Rufino et al., 2008). Hence, lesser food resources in SG suggest that there could be a stronger viability selection on Singapore populations. On its own, this should lead to both males and females being smaller since viability selection acts on both sexes. However, fewer resources could also lead to greater intraspecific competition, especially between males competing over access to resources in order to gain access to potential mates. The intensity of sexual selection on males could be strong enough to counteract the viability selection selecting for smaller body size, thus resulting in extreme male‐biased SSD and males from SG reaching similar sizes to those from MY. Smaller females may produce fewer offspring but will still pass on their genes nonetheless, while smaller males may not even get an opportunity to mate. Body size could thus be such an important trait for males in SG that even under resource limitation, a minimum male body size must be achieved to even stand a chance in finding and securing a mate.

Alternative hypotheses to resource limitation that could affect body size and SSD differences between populations include environmental differences (Dury et al., 2020), differences in predation/parasitism (Servín‐Pastor et al., 2021), differences in gut microbiota due to differing dung resource (Winfrey & Sheldon, 2021), and the possible involvement of cryptic species. Due to the close geographical proximity of peninsular Malaysia and Singapore, most climatic variables such as rainfall and temperature do not significantly differ, with both countries subject to similar patterns of monsoon seasons. A previous study including sites from MY and SG also did not find environmental variables such as temperature and humidity to significantly affect differences in species diversity between SG and MY (Abdul Rahman et al., 2021). As for possible cryptic species, *O. babirussa* is morphologically and molecularly distinct in SG based on our barcoding results. A similar looking but molecularly distinct species, *Onthophagus rufiobscurior*, exists in the forests of MY, but can still be easily discriminated from *O. babirussa* with some taxonomic training. Future work could take into account parasite loads and sequencing of the gut microbiome to investigate them as possible factors differing between populations.

Our results and the above discussion cover potential ultimate forces such as viability and sexual selection and how they could mediate differences in body size. Equally crucial factors to examine are potential proximate mechanisms driving these differences (Beckers et al., 2015). Based on our current findings, it is impossible to tell if the larger male body sizes in SG populations are due to genetic or environmental effects, such as differential gene expression or differential maternal resource partitioning to offspring of different sexes. To investigate the presence of biased maternal investment based on offspring sex, common garden experiments can be carried out by rearing wild‐caught females and testing whether more dung resource is allocated in the construction of brood balls of male larvae. Resource availability is known to affect developmental time and adult body size in *Onthophagus*, with larvae that were allocated fewer resources metamorphosizing earlier and into adults of smaller body size (Shafiei et al., 2001). If mothers from SG populations allocate more dung in the construction of male offspring brood balls than that of females, sex‐biased differential maternal investment in offspring could be the driving proximate mechanism of male‐biased SSD. If no significant differences are found in maternal investment, it is likelier that there is a genetic component such as differential gene expression between the sexes at play.

This study has shown that based on differing degrees of SSD across the populations, it is likely that selection pressures are different between SG and MY. However, in the wild there are multiple sources of selection pressure, and we are unable to pinpoint these sources and their effects here. Future work using quantitative genetics or common garden experiments with manipulated resources could shed more light on the effects of ecology on sexual selection in this species.

### 4.2 Investment in precopulatory and postcopulatory traits varied among populations

Sexual selection can occur before copulation, where males invest in precopulatory traits to increase mating opportunities and after copulation, where males invest in postcopulatory traits to increase chances of fertilizing the ova of females (Birkhead & Pizzari, 2002; Eberhard et al., 2018). Our results show that all four populations showed strong positive static allometry for horn length where horns are disproportionately longer in larger individuals. In dung beetles, horns are weapons used in male–male combat to gain access to breeding females, strong precopulatory sexual selection on horns could explain the strong positive static allometry in male *O. babirussa* (Emlen et al., 2007; Simmons & Ridsdill‐Smith, 2011). Furthermore, compared with the allometric coefficient of classic case studies of sexually selected traits such as deer antlers (*β* = 0.99; Plard et al., 2011), the allometric coefficient for male *O. babirussa* horns was approximately 10‐fold (Figure 5a, *β* = 8.552–9.685), further suggesting the presence of strong precopulatory sexual selection on horns (Kodric‐Brown et al., 2006).

Despite the importance of possessing larger horns in gaining access to females, males with small body sizes and small horns were still regularly sampled and seem to persist in wild populations (Figure 5a). Besides common underlying causes for smaller body and horn size such as food limitation and larval competition, small‐horned males of many *Onthophagus* species utilize alternative mating strategies in which they masquerade as females to sneak past guarding males with larger horns to gain access to breeding females (Beckers et al., 2017; Moczek & Emlen, 2000; Simmons & Ridsdill‐Smith, 2011). Such an alternative mating strategy may exist in *O. babirussa*, which could explain the phenotypic variation in horn length observed in wild‐caught populations (Moczek & Emlen, 2000).

Due to limited resources for growth and development, there may potentially be trade‐offs in the investment of precopulatory and postcopulatory traits (Moczek & Nijhout, 2004). As there was a high relative investment in horn length, a precopulatory trait, we hypothesized that there would be a low relative investment in postcopulatory traits such as testes weight and sperm length. We would also then expect a lower allometric coefficient compared with horn length allometry. However, our results do not show a clear relationship between body size and both testes weight and sperm length across all populations. Looking at the data separated by minor and major morphs (Figure 5d,f), however, some trends can be observed. Testes weight for minor morphs showed a negative allometry, while major morphs showed slight hyperallometry. This could show morph‐specific investment in postcopulatory traits, with minor morphs prioritizing investment in precopulatory traits, while major morphs can afford to invest in postcopulatory traits. This is supported by the much greater horn length allometric coefficient observed in minor males relative to major males (Figure 5b). Sperm length for both morphs was hypoallometric, but major males also showed a slightly steeper allometry and thus more relative investment in this postcopulatory trait. Overall, our findings suggest that investment in horns is more important, suggesting a lower relative investment in sperm length and testes weight than horns, which could be due to weaker postcopulatory selection in male *O. babirussa*. Horns could be so important for mate acquisition that smaller, minor males prioritize investment in horns at the expense of postcopulatory investment, while major males could be at a comfortable horn size threshold required for male–male competition success and thus afford to invest more in postcopulatory traits. To test this, further studies would be needed to identify the rates of polyandry in wild‐caught *O. babirussa* populations by determining paternity estimates of offspring to determine the intensity of postcopulatory sexual selection via sperm competition (McCullough et al., 2017).

It is also interesting to note that House and Simmons (2007) showed that in *Onthophagus taurus*, horn length allometry varied significantly with dung resource quality, while male genitalia exhibited lower allometric slopes than both horns and nonsexual traits, with no clear relationship with dung quality. Conducting similar condition dependence experiments by rearing lines of *O. babirussa* with dung from different species based on mammal diversity differences between the population sites could shed more light on proximate mechanisms driving the difference in relative investment in pre‐ and postcopulatory traits in the species.

## 5 CONCLUSIONS

This study reports population‐level differences in SSD in the species *Onthophagus babirussa*. Populations with lower mammal diversity showed higher degrees of male‐biased SSD, suggesting the importance of dung resource availability and diversity in driving sexual selection. Extreme male‐biased SSD in Singapore populations could be due to higher sexual selection pressure on males outweighing viability selection in females. This is further supported by results showing significant investment in weapons used for competition between males of the species and its relative importance in contrast to postcopulatory traits, which show no clear scaling relationships with body size. These results present an interesting case study, but further studies should be conducted to investigate ultimate forces and proximate mechanisms driving these selection pressures and population level variation.

## AUTHOR CONTRIBUTIONS

**Kai Xin Toh:** Conceptualization (equal); data curation (lead); formal analysis (lead); investigation (equal); methodology (equal); writing – original draft (lead); writing – review and editing (equal). **Sean Yap:** Conceptualization (equal); data curation (supporting); formal analysis (equal); funding acquisition (supporting); investigation (equal); methodology (equal); supervision (supporting); writing – original draft (supporting); writing – review and editing (lead). **Thary Gazi Goh:** Methodology (supporting); project administration (supporting); resources (equal). **Nalini Puniamoorthy:** Conceptualization (equal); funding acquisition (lead); investigation (supporting); project administration (lead); resources (equal); supervision (lead); writing – review and editing (equal).

## CONFLICT OF INTEREST

All authors certify that they have NO affiliations with or involvement in any organization or entity with any financial interest (such as honoraria; educational grants; participation in speakers' bureaus; membership, employment, consultancies, stock ownership, or other equity interest; and expert testimony or patent‐licensing arrangements), or non‐financial interest (such as personal or professional relationships, affiliations, knowledge or beliefs) in the subject matter or materials discussed in this manuscript.

## Data Availability

The R scripts and data that support the findings of this study are openly available in Dryad at Yap, Sean; Toh, Kai Xin (2022), *Onthophagus babirussa* sexual size dimporphism and male sexual trait files and R codes, Dryad, Dataset, https://doi.org/10.5061/dryad.t1g1jwt5d.

## APPENDIX 1

### Field sampling and trap design

**TABLE A1:** Summary of sampling site locations. Sampling in Singapore was conducted with the permission of the National Parks Board, under permit numbers NP/RP18‐034c and NP/RP18‐034‐1. Malaysian specimens were collected with the help of Thary Gazi Goh from the University of Malaya, and sampling was conducted in unprotected forests that do not require permits.

Sampling site	Country	Coordinates
Mandai	Singapore	1.407° N, 103.783° E
		1.400° N, 103.777° E
Chestnut Nature Park	Singapore	1.376° N, 103.782° E
Rifle Range	Singapore	1.355° N, 103.799° E
Windsor Nature Park	Singapore	1.359° N, 103.826° E
Pulau Ubin	Singapore	1.412° N, 103.957° E
Langkawi	Malaysia	6.433° N, 99.708° E
Kenyir	Malaysia	4.962° N, 102.812° E
Temenggor	Malaysia	5.539° N, 101.328° E
Gombak	Malaysia	3.324° N, 101.752° E

**TABLE A2:** Summary of mammal diversity across the four study sites, separated into functional groups determined by size and consumer type

	CCNR	Pulau Ubin	Peninsular Malaysia	Langkawi
Large Herbivore	2	0	7	0
Large Carnivore	1	1	5	1
Large Omnivore	1	1	3	1
Medium Herbivore	5	1	11	4
Medium Carnivore	3	3	10	4
Medium Omnivore	8	3	15	7
Medium Insectivore	1	1	2	1
Small Herbivore	1	0	9	1
Small Carnivore	1	1	4	1
Small Omnivore	13	7	46	16
Small Insectivore	2	1	10	2
Total	38	19	122	38

**TABLE A3:** List of non‐volant mammals present in the four study sites (SG, mainland Singapore; PU, Pulau Ubin; MY, mainland Peninsular Malaysia; LW, Langkawi). Bats were excluded as information about dung beetle association with bat guano is lacking

No.	Order	Family	Genus	Species	Size	Diet	SG	PU	MY	LW
1	Artiodactyla	Bovidae	*Bos*	*gaurus*	Large	Hervbivore			✓	
2	Artiodactyla	Bovidae	*Bos*	*javanicus*	Large	Herbivore			✓	
3	Artiodactyla	Bovidae	*Capricornis*	*sumatraensis*	Large	Hervbivore			✓	
4	Artiodactyla	Cervidae	*Muntiacus*	*muntjak*	Large	Hervbivore	✓		✓	
5	Artiodactyla	Cervidae	*Rusa*	*unicolor*	Large	Hervbivore	✓		✓	
6	Artiodactyla	Suidae	*Sus*	*barbatus*	Large	Omnivore			✓	
7	Artiodactyla	Suidae	*Sus*	*scrofa*	Large	Omnivore	✓	✓	✓	✓
8	Artiodactyla	Tragulidae	*Tragulus*	*kanchil*	Medium	Hervbivore	✓		✓	✓
9	Artiodactyla	Tragulidae	*Tragulus*	*napu*	Medium	Hervbivore	✓	✓	✓	✓
10	Carnivora	Canidae	*Canis*	*familiaris*	Large	Carnivore	✓	✓	✓	✓
11	Carnivora	Canidae	*Cuon*	*alpinus*	Large	Carnivore			✓	
12	Carnivora	Felidae	*Catopuma*	*temminckii*	Medium	Carnivore			✓	
13	Carnivora	Felidae	*Felis*	*catus*	Small	Carnivore	✓	✓	✓	✓
14	Carnivora	Felidae	*Neofelis*	*nebulosa*	Large	Carnivore			✓	
15	Carnivora	Felidae	*Panthera*	*pardus*	Large	Carnivore			✓	
16	Carnivora	Felidae	*Panthera*	*tigris*	Large	Carnivore			✓	
17	Carnivora	Felidae	*Pardofelis*	*marmorata*	Medium	Carnivore			✓	✓
18	Carnivora	Felidae	*Prionailurus*	*bengalensis*	Medium	Carnivore	✓	✓	✓	
19	Carnivora	Felidae	*Prionailurus*	*planiceps*	Medium	Carnivore			✓	
20	Carnivora	Herpestidae	*Urva*	*brachyura*	Small	Carnivore			✓	
21	Carnivora	Herpestidae	*Urva*	*javanica*	Small	Carnivore			✓	
22	Carnivora	Herpestidae	*Urva*	*urva*	Small	Carnivore			✓	
23	Carnivora	Mustelidae	*Aonyx*	*cinereus*	Medium	Carnivore	✓	✓	✓	✓
24	Carnivora	Mustelidae	*Lutra*	*sumatrana*	Medium	Carnivore			✓	✓
25	Carnivora	Mustelidae	*Lutrogale*	*perspicillata*	Medium	Carnivore	✓	✓	✓	✓
26	Carnivora	Mustelidae	*Martes*	*flavigula*	Medium	Omnivore			✓	
27	Carnivora	Mustelidae	*Mustela*	*nudipes*	Medium	Carnivore			✓	
28	Carnivora	Prionodontidae	*Prionodon*	*linsang*	Medium	Carnivore			✓	
29	Carnivora	Ursidae	*Helarctos*	*malayanus*	Large	Omnivore			✓	
30	Carnivora	Viverridae	*Arctictis*	*binturong*	Medium	Omnivore			✓	
31	Carnivora	Viverridae	*Arctogalidia*	*trivirgata*	Medium	Omnivore	✓		✓	✓
32	Carnivora	Viverridae	*Cynogale*	*bennettii*	Medium	Carnivore			✓	
33	Carnivora	Viverridae	*Hemigalus*	*derbyanus*	Medium	Insectivore			✓	
34	Carnivora	Viverridae	*Paguma*	*larvata*	Medium	Omnivore	✓		✓	
35	Carnivora	Viverridae	*Paradoxurus*	*musangus*	Medium	Omnivore	✓	✓	✓	✓
36	Carnivora	Viverridae	*Viverra*	*megaspila*	Medium	Omnivore			✓	
37	Carnivora	Viverridae	*Viverra*	*tangalunga*	Medium	Omnivore	✓		✓	✓
38	Carnivora	Viverridae	*Viverra*	*zibetha*	Medium	Omnivore	✓		✓	
39	Carnivora	Viverridae	*Viverricula*	*indica*	Medium	Omnivore			✓	
40	Dermoptera	Cynocephalidae	*Galeopterus*	*variegatus*	Medium	Hervbivore	✓		✓	✓
41	Eulipotyphla	Erinaceidae	*Echinosorex*	*gymnura*	Small	Insectivore			✓	
42	Eulipotyphla	Erinaceidae	*Hylomys*	*suillus*	Small	Insectivore			✓	
43	Eulipotyphla	Soricidae	*Chimarrogale*	*hantu*	Small	Insectivore			✓	
44	Eulipotyphla	Soricidae	*Crocidura*	*malayana*	Small	Insectivore	✓		✓	
45	Eulipotyphla	Soricidae	*Suncus*	*malayanus*	Small	Insectivore			✓	
46	Eulipotyphla	Soricidae	*Suncus*	*murinus*	Small	Insectivore	✓	✓	✓	✓
47	Eulipotyphla	Taplidae	*Euroscaptor*	*malayana*	Small	Insectivore			✓	
48	Perissodactyla	Tapiridae	*Tapirus*	*indicus*	Large	Hervbivore			✓	
49	Pholidota	Manidae	*Manis*	*javanica*	Medium	Insectivore	✓	✓	✓	✓
50	Primates	Cercopithecidae	*Macaca*	*arctoides*	Medium	Omnivore			✓	
51	Primates	Cercopithecidae	*Macaca*	*fascicularis*	Medium	Omnivore	✓	✓	✓	✓
52	Primates	Cercopithecidae	*Macaca*	*nemestrina*	Medium	Omnivore			✓	✓
53	Primates	Cercopithecidae	*Presbytis*	*femoralis*	Medium	Hervbivore	✓		✓	
54	Primates	Cercopithecidae	*Presbytis*	*robinsoni*	Medium	Hervbivore			✓	
55	Primates	Cercopithecidae	*Presbytis*	*siamensis*	Medium	Hervbivore			✓	
56	Primates	Cercopithecidae	*Trachypithecus*	*cristatus*	Medium	Hervbivore			✓	
57	Primates	Cercopithecidae	*Trachypithecus*	*obscurus*	Medium	Hervbivore	✓		✓	✓
58	Primates	Hylobatidae	*Hylobates*	*agilis*	Medium	Hervbivore			✓	
59	Primates	Hylobatidae	*Hylobates*	*lar*	Medium	Hervbivore			✓	
60	Primates	Hylobatidae	*Symphalangus*	*syndactylus*	Medium	Hervbivore			✓	
61	Primates	Lorisidae	*Nycticebus*	*coucang*	Medium	Omnivore	✓		✓	✓
62	Proboscidea	Elephantidae	*Elephas*	*maximus*	Large	Hervbivore			✓	
63	Rodentia	Hystricidae	*Atherurus*	*macrourus*	Medium	Omnivore			✓	
64	Rodentia	Hystricidae	*Hystrix*	*brachyura*	Medium	Omnivore	✓	✓	✓	✓
65	Rodentia	Hystricidae	*Trichys*	*fasciculata*	Small	Herbivore			✓	
66	Rodentia	Muridae	*Bandicota*	*bengalensis*	Small	Omnivore			✓	
67	Rodentia	Muridae	*Bandicota*	*indica*	Small	Omnivore			✓	
68	Rodentia	Muridae	*Berylmys*	*bowersi*	Small	Omnivore			✓	
69	Rodentia	Muridae	*Chiropodomys*	*gliroides*	Small	Herbivore			✓	
70	Rodentia	Muridae	*Lenothrix*	*canus*	Small	Omnivore			✓	
71	Rodentia	Muridae	*Leopoldamys*	*edwardsi*	Small	Omnivore			✓	
72	Rodentia	Muridae	*Leopoldamys*	*sabanus*	Small	Omnivore			✓	✓
73	Rodentia	Muridae	*Maxomys*	*inas*	Small	Omnivore			✓	
74	Rodentia	Muridae	*Maxomys*	*rajah*	Small	Omnivore	✓		✓	✓
75	Rodentia	Muridae	*Maxomys*	*surifer*	Small	Omnivore			✓	✓
76	Rodentia	Muridae	*Maxomys*	*whiteheadi*	Small	Omnivore			✓	✓
77	Rodentia	Muridae	*Mus*	*caroli*	Small	Omnivore			✓	
78	Rodentia	Muridae	*Mus*	*musculus*	Small	Omnivore	✓	✓	✓	
79	Rodentia	Muridae	*Niviventer*	*bukit*	Small	Omnivore			✓	
80	Rodentia	Muridae	*Niviventer*	*cremoriventer*	Small	Omnivore			✓	✓
81	Rodentia	Muridae	*Niviventer*	*fulvescens*	Small	Omnivore			✓	
82	Rodentia	Muridae	*Pithecheir*	*parvus*	Small	Omnivore			✓	
83	Rodentia	Muridae	*Rattus*	*argentiventer*	Small	Omnivore			✓	
84	Rodentia	Muridae	*Rattus*	*exulans*	Small	Omnivore	✓	✓	✓	✓
85	Rodentia	Muridae	*Rattus*	*norvegicus*	Small	Omnivore	✓		✓	✓
86	Rodentia	Muridae	*Rattus*	*tanezumi*	Small	Omnivore	✓	✓	✓	✓
87	Rodentia	Muridae	*Rattus*	*tiomanicus*	Small	Omnivore	✓	✓	✓	✓
88	Rodentia	Muridae	*Sundamys*	*annandalei*	Small	Omnivore	✓	✓	✓	
89	Rodentia	Muridae	*Sundamys*	*muelleri*	Small	Omnivore			✓	✓
90	Rodentia	Sciuridae	*Aeromys*	*tephromelas*	Small	Hervbivore			✓	
91	Rodentia	Sciuridae	*Callosciurus*	*caniceps*	Small	Omnivore			✓	✓
92	Rodentia	Sciuridae	*Callosciurus*	*erythraeus*	Small	Omnivore			✓	
93	Rodentia	Sciuridae	*Callosciurus*	*nigrovittatus*	Small	Omnivore			✓	
94	Rodentia	Sciuridae	*Callosciurus*	*notatus*	Small	Omnivore	✓	✓	✓	✓
95	Rodentia	Sciuridae	*Callosciurus*	*prevostii*	Small	Omnivore			✓	
96	Rodentia	Sciuridae	*Dremomys*	*rufigenis*	Small	Omnivore			✓	
97	Rodentia	Sciuridae	*Hylopetes*	*sagitta*	Small	Omnivore			✓	
98	Rodentia	Sciuridae	*Hylopetes*	*spadiceus*	Small	Omnivore	✓		✓	✓
99	Rodentia	Sciuridae	*Iomys*	*horsfieldii*	Small	Omnivore	✓		✓	
100	Rodentia	Sciuridae	*Lariscus*	*insignis*	Small	Omnivore			✓	
101	Rodentia	Sciuridae	*Petaurillus*	*kinlochii*	Small	Omnivore			✓	
102	Rodentia	Sciuridae	*Petaurista*	*elegans*	Small	Hervbivore			✓	
103	Rodentia	Sciuridae	*Petaurista*	*petaurista*	Small	Herbivore	✓		✓	✓
104	Rodentia	Sciuridae	*Petinomys*	*genibarbis*	Small	Omnivore			✓	
105	Rodentia	Sciuridae	*Petinomys*	*setosus*	Small	Omnivore			✓	
106	Rodentia	Sciuridae	*Petinomys*	*vordermanni*	Small	Omnivore			✓	
107	Rodentia	Sciuridae	*Pteromyscus*	*pulverulentus*	Small	Hervbivore			✓	
108	Rodentia	Sciuridae	*Ratufa*	*affinis*	Small	Herbivore			✓	
109	Rodentia	Sciuridae	*Ratufa*	*bicolor*	Small	Omnivore			✓	✓
110	Rodentia	Sciuridae	*Rhinosciurus*	*laticaudatus*	Small	Omnivore	✓		✓	
111	Rodentia	Sciuridae	*Sundasciurus*	*hippurus*	Small	Omnivore			✓	
112	Rodentia	Sciuridae	*Sundasciurus*	*lowii*	Small	Omnivore			✓	
113	Rodentia	Sciuridae	*Sundasciurus*	*tenuis*	Small	Omnivore	✓		✓	✓
114	Rodentia	Sciuridae	*Tamiops*	*mcclellandii*	Small	Omnivore			✓	
115	Rodentia	Spalacidae	*Rhizomys*	*pruinosus*	Small	Herbivore			✓	
116	Rodentia	Spalacidae	*Rhizomys*	*sumatrensis*	Small	Hervbivore			✓	
117	Scandentia	Ptilocercidae	*Ptilocercus*	*lowii*	Small	Omnivore			✓	
118	Scandentia	Tupaiidae	*Tupaia*	*glis*	Small	Omnivore	✓	✓	✓	✓
119	Scandentia	Tupaiidae	*Tupaia*	*minor*	Small	Omnivore			✓	
120	Soricomorpha	Soricidae	*Crocidura*	*attenuata*	Small	Insectivore			✓	
121	Soricomorpha	Soricidae	*Crocidura*	*fuliginosa*	Small	Insectivore			✓	✓
122	Soricomorpha	Soricidae	*Crocidura*	*monticola*	Small	Insectivore			✓	

Sources: Balakirev, A. E., Abramov, A. V. & Rozhnov, V. V. (2011). Taxonomic revision of Niviventer (Rodentia, Muridae) from Vietnam: a morphological and molecular approach. Russian Journal of Theriology, 10(1), 1–26. Davison, G. W. H. & Zubaid, A. (2007). The status of mammalian biodiversity in Malaysia. Status of Biological Diversity in Malaysia and Threat Assessment of Plant Species in Malaysia. Kuala Lumpur: Forest Research Institute Malaysia, 3–27. Hinckley, A., Camacho‐Sanchez, M., Ruedi, M., Hawkins, M.T., Mullon, M., Cornellas, A., Tuh Yit Yuh, F. & Leonard, J.A. (2021). Evolutionary history of Sundaland shrews (Eulipotyphla: Soricidae: Crocidura) with a focus on Borneo. Zoological Journal of the Linnean Society. Meijaard, E. (2003). Mammals of south‐east Asian islands and their Late Pleistocene environments. Journal of Biogeography, 30(8), 1245–1257. National Parks Board. (2021). Checklist of mammals from Singapore. Terrestrial and Marine Mammals. Retrieved from https://www.nparks.gov.sg/biodiversity/wildlife‐in‐singapore/species‐list/mammal. National Parks Board. (2014). Checklist of Mammals of Pulau Ubin. Pulau Ubin. Retrieved from https://www.nparks.gov.sg/~/media/nparks‐real‐content/gardens‐parks‐and‐nature/parks‐and‐nature‐reserve/pulau‐ubin/documents/checklist‐mammals.ashx?la=en Rufino, M. B. M., Magintan, D., Ngau, C., Ismail, A. Z., Jamaludin, H., Zainal, A. M., Rasdi, I., Hashim, A.K.A., Ten, D.C.Y. & Fauzul Azim, Z. A. (2008). Mammals of Temenggor Forest Reserve: Evidence through camera trapping. In Proceedings of the National Biodiversity Seminar (pp. 7–16).

## APPENDIX 2

### Morphological and molecular sorting of *Onthophagus babirussa*

#### Morphological sorting

*Onthophagus babirussa* were separated from other species via their distinct thorax patterning. Specifically, the lateral portions on both sides of the thorax are in a lighter shade of brown compared to rest of thorax and contain one or two darker brown spots (Figures S2 and S3). Mid femurs of all individuals were dissected for molecular work (see below) and male *O*. *babirussa* individuals were separated from females based on the presence of head horns. Male individuals were kept in separate, labelled Eppendorf tubes with a drop of 1× Phosphate‐Buffered solution (PBS).

### Molecular sorting and investigating genetic variation

DNA was extracted from 739 specimens from Singapore populations (CCNR = 129 and Pulau Ubin = 167) and Malaysian populations (Central Peninsular MY = 109 and Langkawi = 334). The cytochrome oxidase I gene (COI) fragment has been widely used as to distinguish and identify species for most animals as the mutation rate of COI gene is approximately parallel to speciation time scale, thus able to differentiate between species that are closely related (Fraija‐Fernández et al., 2018; Hebert et al., 2003; Waugh, 2007). Furthermore, the COI gene is an ideal species marker for insects due to the simple sequence alignment, primer sites that are robust and easily available and low likelihood of recombination and possession of introns (Foottit & Adler, 2009). A 3% threshold for uncorrected pairwise distances was used as it is commonly used in literature to differentiate species (Hebert et al., 2003; Meiklejohn et al., 2011; Srivathsan & Meier, 2012).

For all specimens, the right mid femur was dissected into 7 μl of QuickExtract solution and the DNA was extracted by following Lucigen's (the manufacturer's) protocol (Lucigen, 2018). After DNA extraction, COI amplification was conducted on extracted DNA. The reaction volumes for COI amplification for each sample was 5 μl of CWBIO 2xTaq MasterMix (Dye), 1 μl of extracted DNA, 1 μl of sterilised Millipore water, 1 μl of forward primer, “mlCOIintF”: 5′‐ GGW ACW GGW TGA ACW GTW TAY CCY CC‐3′ (Leray et al., 2013), 1 μl of reverse primer, “jgHCO2198”: 5′‐TAN ACY TCN GGR TGN CCR AAR AAY CA‐3′ (Geller et al., 2013) and 1 μl of 0.001 mg Bovine Serum Albumin (BSA). Polymerase Chain Reaction (PCR) was performed using the Eppendorf Mastercycler nexus gradient using a step‐up cycling protocol. The following protocol was used: initial denaturation (94°C, 5 min) followed by 35 cycles of denaturation (94°C, 1 min), annealing (47°C, 2 min) and extension (72°C, 1 min) and lastly, final extension (72°C, 5 min). In addition, primers were labelled with a sequence tag of 7–9 bp such that all specimens will have a unique combination of labelled forward and reverse primers. The successfully amplified PCR products were pooled and sent for NGS sequencing, and sequences were analysed by constructing a cluster fusion diagram using uncorrected pairwise distances.

### NGS and sequence analysis

According to the manufacturer's instructions, the PCR products underwent bead cleanup to purify the products. Subsequently, the products were quantified, pooled in equimolar ratios and submitted for library preparation by Genome Institute of Singapore. Then, Next‐Generation Sequencing (NGS) was conducted using MiSeq sequencing platform to obtain 313 bp fragment of COI gene.

Sequence analysis was then conducted with reference to the analysis pipeline detailed by (Meier et al., 2016). The reads from the paired‐ends were merged by the software PEAR 0.9.6 (Zhang et al., 2014). The reads of each PCR products were then matched to their specific template specimen which was achieved due to primer pair combination that were uniquely labelled. A python script by (Srivathsan, unpublished) was used to (1) demultiplex data, (2) tally the reads for each sample, (3) identify and cluster identical reads into groups, (4) identify dominant groups of reads and combine with variants that were otherwise of identical length and lastly (5) tally the reads found in the group showing highest identity and compare with the group showing the next highest identity (Meier et al., 2016). Quality control was carried out by a set of criteria namely more than 50× read count, more than 10× barcode count and for the number of dominant reads to be five times or more than second most dominant reads (Meier et al., 2016). This was to ensure that coverage attributed to each barcode was sufficient and not from confounding sequences such as contaminant DNA fragments. In addition, quality control rejects dominant sequences that may have arisen out of amplification error in the PCR step. Next, the sequences that passed the quality control were entered into the search query in Basic Local Alignment Search Tool (BLAST) to search for sequences that match >97% to non‐Onthophagus taxa, which were contaminant sequences and thus eliminated from analysis. After quality control, MEGA7, an online software, was used to align the sequences to ensure that there were no stop codons. Then, a new Python script (Srivathsan, unpublished) was used to construct a cluster fusion diagram based on uncorrected pairwise distances, and a threshold of 3% was used to delimit species. This 3% pairwise distance threshold is widely used to distinguish between insect species in literature (Hebert et al., 2003; Srivathsan & Meier, 2012). A cluster fusion diagram of a subset of *O. babirussa* haplotypes from across the sampling sites is shown in Figure A4 below. The cluster fusion shows that there is little geographic pattern in the distribution of haplotypes, and that all haplotypes fall under the 3% pairwise distance threshold, supporting our morphological sorting of the specimens under the same species.
